# Morphometric Parameters of the Ear and Their Sexual Dimorphism in the Eastern Province of Saudi Arabia

**DOI:** 10.7759/cureus.51625

**Published:** 2024-01-03

**Authors:** Sanket D Hiware, Khaled A AlFaqeeh, Salim S Alattallah, Abdullah Y Faisal, Abdullah M Alessa, V. Christopher Amalraj, Essam E Ismail, Sujatha B Bayer, Brij Raj Singh, Ujwal Gajbe

**Affiliations:** 1 Anatomy, Graphic Era Institute of Medical Sciences, Dehradun, IND; 2 Anatomy, College of Medicine, Imam Abdulrahman Bin Faisal University, Dammam, SAU; 3 Development and Community, College of Medicine, Imam Abdulrahman Bin Faisal University, Dammam, SAU; 4 Anatomy, Datta Meghe Medical College, Datta Meghe Institute of Higher Education and Research, Wardha, IND

**Keywords:** ear height, ear width, antihelix of the ear, helix of the ear, tragus of the ear, ear anatomy, ear anthropometry, human ear, sexual dimorphism, ear morphometry

## Abstract

Background

The human ear is a distinctive facial feature, revealing valuable information about ethnicity, gender, and age. Anthropometric measures play a crucial role in fields such as forensic medicine, prosthetics, and plastic surgery. The external ear, known for its diversity in structure and individual characteristics, has become a subject of interest in various populations. This study aims to determine the mean values of morphometric measurements for both right and left ears while exploring sexual dimorphism in ear dimensions.

Methodology

A prospective, observational, cross-sectional study was conducted among 200 volunteers from the eastern province of Saudi Arabia, comprising 98 males and 102 females, at the Department of Anatomy, Institute of Medical Sciences, Imam Abdulrahman Bin Faisal University. Participants were randomly selected from King Fahad University Hospital workers and patients. Using a Vernier caliper and after obtaining consent, various aspects of ear morphology were measured. The study employed statistical analyses such as the volunteers’ t-test, Pearson’s coefficient of correlation, and linear regression equations.

Results

In males, the mean total height of the right and left ears was 6.054 ± 0.5394 and 6.044 ± 0.5235 cm, respectively, while for females, it was 5.489 ± 0.4481 and 5.763 ± 4.8446 cm, respectively. The mean widths, heights, and other dimensions of the ears exhibited variations between genders.

Conclusions

The study provides comprehensive insights into the dimensions and indices of the pinna among the population of the eastern province of Saudi Arabia. The findings confirmed the presence of sexual dimorphism in the ear measurements, consistent with observations in other ethnic groups.

## Introduction

Anthropometry involves examining human physical diversity by measuring the proportions of living bodies. The anthropometry of the human ear has been studied for many years. The morphometric parameters of the ear can be useful in fields such as forensic medicine, prosthetics, and plastic surgery [1,2]. Ear malformations account for half of the cases in the ear, nose, and throat (ENT) region. These malformations are generally unilateral and can be acquired, familial, genetic, or associated with syndromes. A precise understanding of these malformations along with the expected normal morphology of the ear can be very helpful in planning the overall treatment and rehabilitation of the patient [3].

The external human ear, known for its diverse structure, exhibits variations in both physical and individual traits among different individuals and groups. Remarkably, the ear stands out as the most distinctive facial feature, offering insights into ethnicity, gender, and age. Its size, shape, and position on the face are aesthetically significant. Globally, studies have revealed variations in the morphology and measurements of human ears. Recent research indicates that each component of the outer ear possesses a unique shape, varying among individuals and groups [4-7]. These studies have classified different ear features, including helix and tragus shapes, Darwin’s tubercles, earlobe characteristics, and their prevalence in diverse populations. Metrics such as linear distances, ratios, growth changes, and bilateral asymmetry have been scrutinized to establish population and community profiles for forensic and anthropological purposes. These investigations underscore significant differences in ear sizes between individuals and populations, with males generally exhibiting larger ear parameters than females [5-13]. This study aimed to establish the normal range of some morphometric parameters of the ear and analyze their sexual dimorphism.

## Materials and methods

This cross-sectional study involved 200 participants from the eastern province of the Kingdom of Saudi Arabia. Individuals displaying ear deformities, masses, congenital malformations, or severe ear trauma were excluded from the study. The inclusion criteria comprised volunteers aged 20 and above, whose parents were Saudi Arabian citizens. Participants were briefed on the study’s objectives and provided written consent. Study approval was obtained from the Institutional Review Board at Imam Abdulrahman Bin Faisal University (approval number: IRB-UGS-2022-01-482).

Before assessing various ear parameters, demographic information was collected, including age, ethnicity, height, weight, body mass index (BMI), blood type, presence of piercings, and ear lobe type (free or attached). Measurements were conducted on both ears, encompassing lobular height (LH), lobular width (LW), total ear height (TEH), ear width (EW), tragus to helix (TH), tragus to antihelix (TA), ear prominence (EP), and nasion to ear distance.

**Figure 1:**
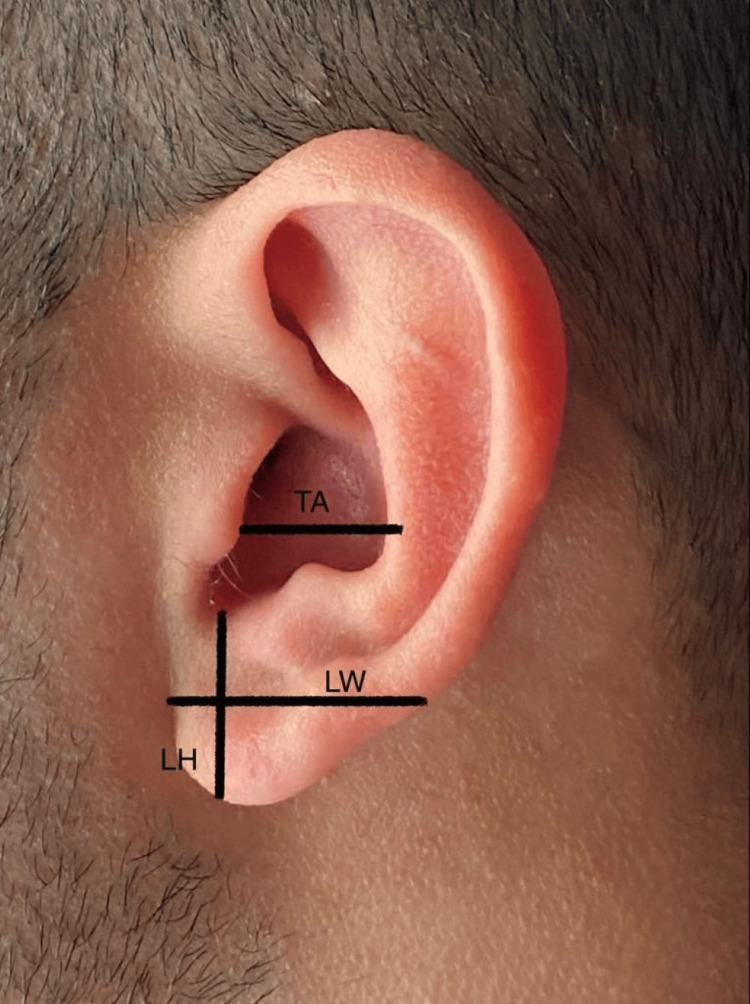
Morphometric measurements of TA, LH, and LW. TA = tragus to antihelix; LH = lobule height; LW = lobule width

**Figure 2:**
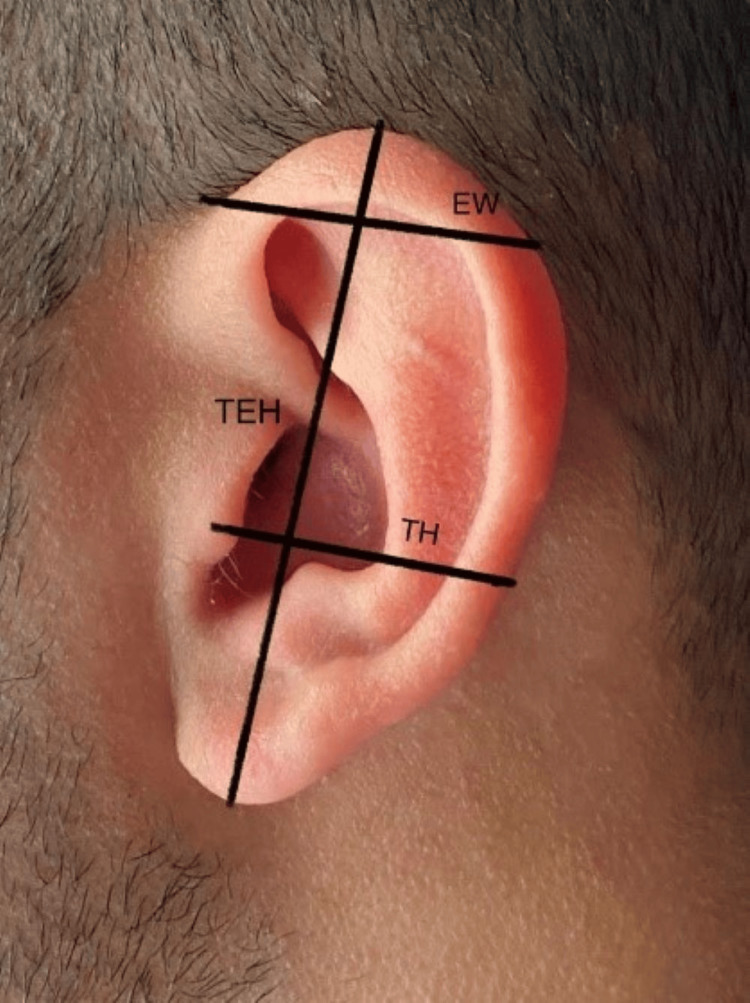
Morphometric measurements of TEH, TH, and EW. TEH = total ear height; TH = tragus to helix; EW = ear width

**Figure 3:**
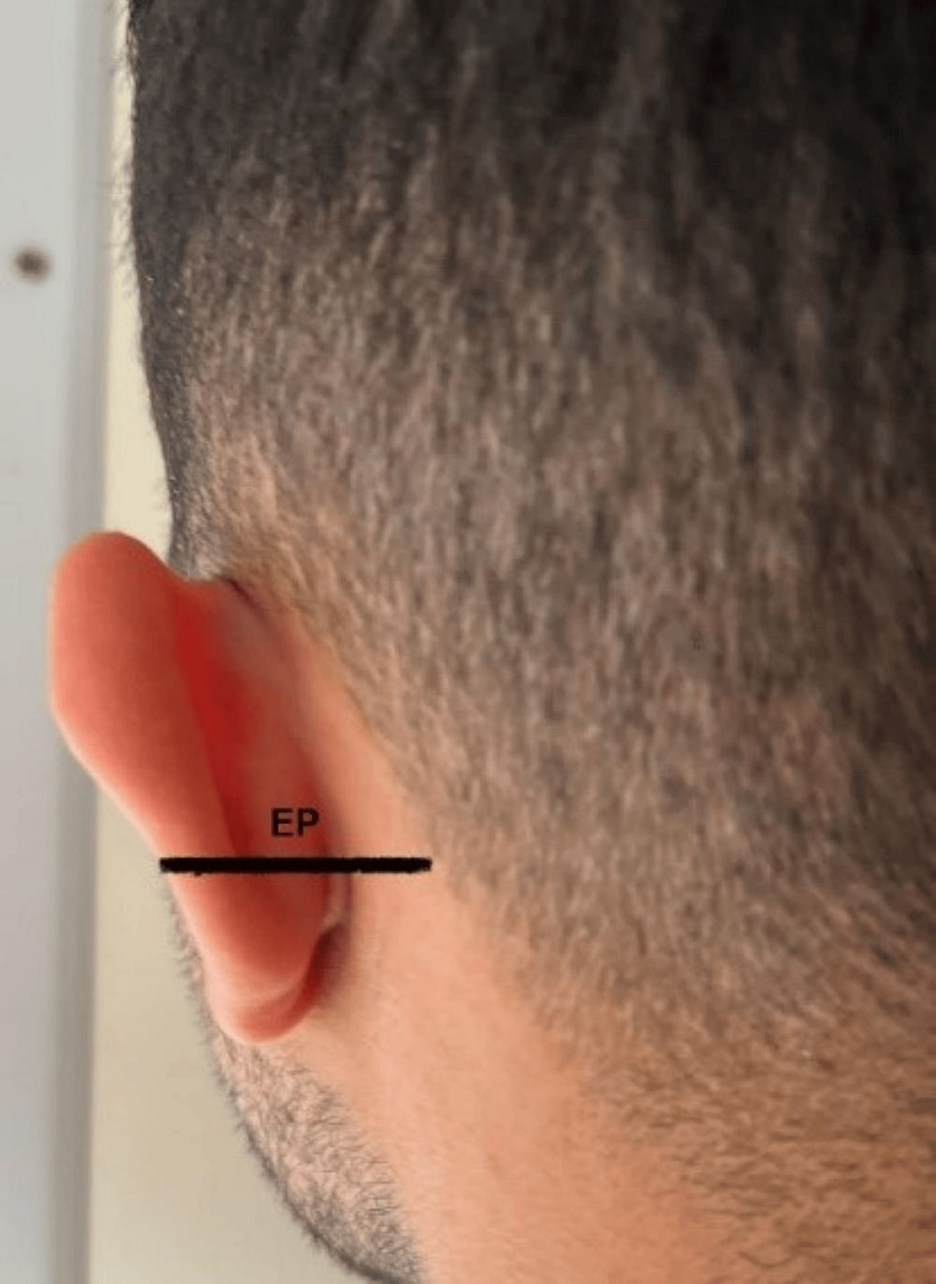
Morphometric measurements of EP. EP = ear prominence

**Figure 4:**
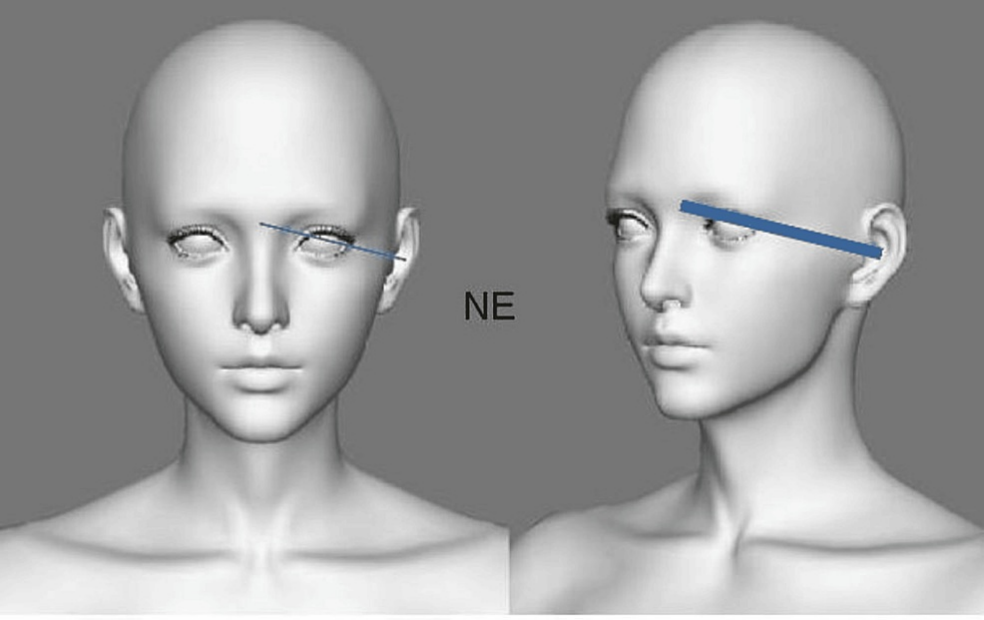
Morphometric measurements of NE. NE = nasion to ear

LH was measured as the distance from the highest point of the lobule to the lowest (Figure 1). LW was determined as the distance from the most medial point of the lobe to the lateral point (Figure 1). TEH represented the measurement between the lobe’s lowest point and the auricle’s highest point (Figure 2). EW denoted the distance from the horizontal root of the helix to the helix (Figure 2). TH was measured (Figure 2) as was TA (Figure 1). EP was the distance from the helix to the mastoid (Figure 3), and the nasion to ear distance was also recorded (Figure 4). All measurements were conducted using a Vernier caliper while volunteers were comfortably seated with a straight back. The recorded measurements were expressed in centimeters.

Statistical analysis

The data were inputted into a Microsoft Excel sheet in 2019, and the analysis was conducted using SPSS version 24.0 (IBM Corp., Armonk, NY, USA). The criterion for statistical significance was established with a p-value below 0.05. This threshold indicated a noteworthy difference. The strength of the correlation was assessed utilizing Pearson’s correlation coefficient to ascertain the degree of association between variables.

## Results

A total of 200 volunteers (98 males and 102 females) were included in the study randomly. The mean age of the male and female participants was 26.10 ± 9.093 years and 23.83 ± 8.062 years, respectively. The mean age of the males and females together was 24.95 ± 8.636 years. The two-tailed p-value was 0.063 (Table 1), which was not statistically significant.

**Table 1:** The number of participants and their mean age. ns = not statistically significant (p > 0.05)

Parameters	Male, N (%)	Female, N (%)	Total, N (%)	P-value
Number of participants	98 (49)	102 (51)	200 (100)	0.063^ns^
Age (mean ± SD)	26.10 ± 9.093	23.83 ± 8.062	24.95 ± 8.636	

Statistically significant differences were observed between men and women in TEH (p = 0.000), which were found to be 6.054 ± 0.5394 cm and 5.489 ± 0.4481 cm, respectively. Regarding the total height of the left ear, it was not statistically significant (p = 0.569), with the measurements for males and females being 6.044 ± 0.5235 cm and 5.763 ± 4.8446 cm, respectively. Regarding the width of the right ear, no significant difference was observed between men and women (p = 0.862), and the measurements were 3.052 ± 0.5006 cm and 3.038 ± 0.6 160 cm, respectively. Lastly, the widths of the left ear of males and females were statistically significant (p = 0.006), and the measurements were 2.978 ± 0.5110 cm and 3.686 ± 2.4824 cm, respectively (Table 2).

**Table 2:** The comparison between males and females among the sides in the total ear height and ear width. ns = not statistically significant (p > 0.05); * = statistically significant (p < 0.05)

Side	Sex	Total ear height, mean ± SD (cm)	P-value	Ear width, mean ± SD (cm)	P-value
Right	Male	6.054 ± 0.5394	0.000^*^	3.052 ± 0.5006	0.862^ns^
	Female	5.489 ± 0.4481		3.038 ± 0.6160	
Left	Male	6.044 ± 0.5235	0.569^ns^	2.978 ± 0.5110	0.006^*^
	Female	5.763 ± 4.8446		3.686 ± 2.4824	

No statistically significant differences were observed between men and females in the height of the right ear lobes (p = 0.063), which were found to be 1.695 ± 0.3253 cm and 1.749 ± 1.0647 cm, respectively. Regarding the height of the left ear lobule, it was also not statistically significant (p = 0.149), with the measurements for males and women being 1.686 ± 0.3143 cm and 1.884 ± 1.3232 cm, respectively. Compared to the widths of the right ear lobes, a significant difference was observed between men and females (p = 0.000), and the measurements were 1.988 ± 0.3465 cm and 2.297 ± 0.2830 cm, respectively. Lastly, the width of the left ear lobe of males and females was also statistically significant (p = 0.006) and the measurements were 1.964 ± 0.3474 cm and 2.522 ± 1.9687, respectively (Table 3).

**Table 3:** The comparison between males and females among the sides in height and width. ns = not statistically significant (p > 0.05); * = statistically significant (p < 0.05)

Side	Sex	Ear lobule height, mean ± SD (cm)	P-value	Ear lobule width, mean ± SD (cm)	P-value
Right	Male	1.695 ± 0.3253	0.630^ns^	1.988 ± 0.3465	0.000^*^
	Female	1.749 ± 1.0647		2.297 ± 0.2830	
Left	Male	1.686 ± 0.3143	0.149^ns^	1.964 ± 0.3474	0.006^*^
	Female	1.884 ± 1.3232		2.522 ± 1.9687	

Statistically significant differences were not evident in the assessment of TH measurements between males and females. For the right side, the calculated p-value was 0.885, with mean measurements of 2.922 ± 0.3715 cm for males and 2.889 ± 2.2415 cm for females. Similarly, the TH measurements on the left side did not exhibit statistical significance, as indicated by a p-value of 0.898. The mean values for this parameter were 2.890 ± 0.3594 cm for males and 2.860 ± 2.2830 cm for females. Likewise, no statistically significant difference was observed in TA measurements between males and females on the right side (p = 0.182). The mean values for males and females were 1.892 ± 0.3415 cm and 2.045 ± 1.0830 cm, respectively. However, a noteworthy statistical difference emerged on the left side (p = 0.000), with mean measurements of 1.903 ± 0.4045 cm for males and 2.612 ± 1.2913 cm for females (Table 4).

**Table 4:** The comparison between males and females among the sides in the tragus to helix and tragus to antihelix. ns = not statistically significant (p > 0.05); * = statistically significant (p < 0.05)

Side	Sex	Tragus to helix, mean ± SD (cm)	P-value	Tragus to antihelix, mean ± SD (cm)	P-value
Right	Male	2.922 ± 0.3715	0.885^ns^	1.892 ± 0.3415	0.182^ns^
	Female	2.889 ± 2.2415		2.045 ± 1.0830	
Left	Male	2.890 ± 0.3594	0.898^ns^	1.903 ± 0.4045	0.000^*^
	Female	2.860 ± 2.2830		2.612 ± 1.2913	

In the comparison of the mean distance between the right ear and the nasion, males exhibited a measurement of 13.160 ± 0.9715 cm, whereas females had a mean of 12.330 ± 0.5282 cm. This difference was found to be statistically significant (p = 0.000). However, there was no significant difference in the mean distance from the left ear to the nasion in males (13.123 ± 0.9725 cm) and females (13.419 ± 11.0488 cm), with a p-value of 0.972. The analysis of the type of lobule in the right ear showed a mean of 0.51 ± 0.502 cm for males and 0.57 ± 0.498 for females cm, with no significant differences (p = 0.410). Similarly, no significant distinctions were observed in the mean type of the left ear between males and females (p = 0.41) (Table 5).

**Table 5:** The comparison between males and females among the sides in the nasion to ear and lobule type. ns = not statistically significant (p > 0.05); * = statistically significant (p < 0.05)

Side	Sex	Nasion to ear, mean ± SD (cm)	P-value	Lobule type, mean ± SD	P-value
Right	Male	13.160 ± 0.9715	0.000^*^	0.51 ± 0.502	0.410^ns^
	Female	12.330 ± 0.5282		0.57 ± 0.498	-
Left	Male	13.123 ± 0.9725	0.792^ns^	0.51 ± 0.502	0.410^ns^
	Female	13.419 ± 11.0488		0.57 ± 0.498	-

## Discussion

The human ear is an important part of our body with different sizes and shapes. It is a crucial distinguishing characteristic of the face. The question then arises: what is a normal ear? In our study, our objective was to calculate the so-called normal ear anthropometric measurements in the eastern province of Saudi Arabia. Many studies have been conducted among different ethnic groups in Thailand, Sudan, Turkey, and many other countries [4-13], but, to our knowledge, this is the first study among the population of the eastern province of Saudi Arabia. Furthermore, our study demonstrates the anthropometric measurements of the human ear in the eastern province, which is a crucial aspect of many of our lives. For example, plastic surgeons need it for reconstruction surgeries and general surgeons need to know the normal range of different parameters of the human ear to identify congenital abnormalities [14-16].

Forensic practice, anthropology, anatomy, and plastic surgery have all benefited from the information on anthropometric measurements of the ears. In our study, we measured the LH, LW, TEH, EW, TH, TA, EP, and nasion to ear distance, whose significance was described in several tables and charts. We started with the dimensions of the ear lobes, which are crucial in forensics to correlate a specific dimension with a specific sex.

In a previous study conducted in Ghana, both males and females from the Dagaabas people had bilateral asymmetry between the heights of the right ear lobes (1.418 ± 0.207 cm, 1.437 ± 0.250 cm, respectively) and left ear lobule heights (1.435 ± 0.280 cm, 1.471 ± 0.235 cm, respectively). Regarding the widths of the right ear lobes, they were 1.547 ± 0.229 cm and 1.521 ± 0.195 cm, respectively, and the widths of the left ear lobes were 1.563 ± 0.249 cm and 1.544 ± 0.205 cm, respectively [17]. However, a study aimed at the Indian population concluded that the heights were not significantly different between both sexes, but the lobule widths of the right and left ears were significantly higher in males [18]. Similarly, a study from Turkey showed that the measurements of the heights of the right and left lobes in males (1.84 ± 0.17 cm, 1.83 ± 0.17 cm, respectively) were higher than in women (1.79 ± 0.15 cm, 1.75 ± 0.14 cm, respectively), and the widths of the right and left ear lobule widths in men (1.98 ± 0.19 cm, 1.94 ± 0.2 cm, respectively) were also higher than in females (1.89 ± 0.2 cm, 1.85 ± 0.22 cm, respectively) [19]. These measurements are similar to a study done among Sudanese Arabs as well [20]. In the Thai population, the height of the left ear lobe was found to be higher in females (1.94 ± 0.39 cm) than in males (1.86 ± 0.36 cm). However, the widths of the right and left lobes and the right lobule heights were greater in males [15].

In our study, we found that the measurements of the widths of the right and left ear lobes of females (2.297 ± 0.283 cm, 2.522 ± 1.969 cm, respectively) were significantly higher than in males (1.988 ± 0.347 cm, 1.964 ± 0.347 cm respectively), which could be attributed to normal physiology, genetics, ethnicity, and the environment. However, as mentioned in previous studies, measurements of right and left ear lobes were similar in both sexes. The measurements were 1.695 ± 0.325 cm and 1.686 ± 0.314 cm, respectively, for men and 1.749 ± 1.065 cm and 1.884 ± 1.323 cm, respectively, for females. We concluded that the height of the ear lobule did not correlate with a specific sex, yet the width of the ear lobules in women was almost always significantly different from that of men.

**Figure 5:**
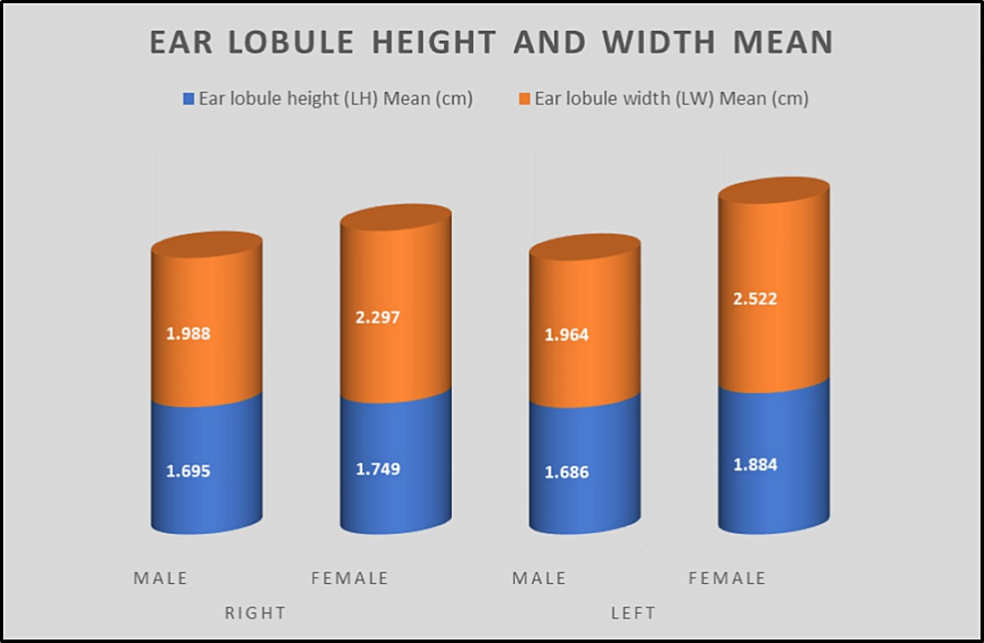
The comparison between males and females for ear lobule height and width of the ear lobe for both sides.

Figure 5 shows the comparison between males and females for ear lobule height and width of the ear lobe for both sides.

Our investigation reveals the potential applications of anthropometric measurements in the field of medicolegal practice. A study conducted in Sudan aimed to demonstrate sexual dimorphism in the morphology of the human external ear, establishing a reliable method for determining the sex associated with a particular ear. Notably, the study found that male ear dimensions were consistently higher than those of females, except for lobular length [20]. Our study also incorporated measurements of TEH and EW, aligning with studies conducted in diverse geographical locations.

In a Turkish study, the total height of the left ear was reported as 6.31 ± 0.36 cm in males and 5.97 ± 0.3 cm in females [19]. Similarly, a study from Thailand yielded measurements of 6.73 cm and 6.36 cm for males and females, respectively [15]. Our investigation revealed that the total height of the left ear in males was 6.044 ± 0.5235 cm, while in females, it was 5.763 ± 0.48446 cm. This aligns closely with the findings from Turkey, supporting the consensus across studies that males tend to exhibit greater TEH, a trend observed in both the Thai and Sudanese Arab populations [14,15,19,20].

In the Turkish population, the total height of the right ear displayed no significant differences between males (6.29 ± 0.35 cm) and females (5.97 ± 0.3 cm) [14]. Conversely, in the Thai population, a significant difference in the same measurement was observed, with values of 6.7 cm for males and 6.3 cm for females [15]. Our study highlighted significant sexual dimorphism in the left ear, with measurements for the right ear in males at 6.054 ± 0.5394 cm and in females at 5.489 ± 0.4481 cm. This pattern is consistent with observations in the Thai and Sudanese populations but contrasts with the non-significant differences reported in the Turkish population [14,15,18,20].

The width of the right ear in the Turkish population between males and females showed no significant differences or sexual dimorphism. The right ear width in males was 3.31± 0.21 cm and that in females was 3.12± 0.22 cm [19]. In the Thai population, there was a significant difference between males and females, with results of 3.46 cm and 3.18 cm, respectively [15]. In our study, we also found no significant differences between males and females, with results of 3.052 ± 0.5006 cm and 3.038 ± 0.6160 cm, respectively, similar to the Turkish population. However, we can see that the Sudanese study found sexual dimorphism in their data, which is consistent with the Thai population [15,20].

The width of the left ear in the Turkish population between males and females showed no sexual dimorphism, with results of 3.33 ± 0.21 cm and 3.13 ± 0.22 cm, respectively [14]. In the Thai population, a significant difference was noted between males and females, with results of 3.49 cm and 3.26 cm, respectively [15]. Moreover, in the Sudanese population, sexual dimorphism was found [20]. Our study agrees with the width of the sexual dimorphism in the left ear, with results of 2.978 ± 0.5110 cm in men and 3.686 ± 2.4824 cm in females. We need to emphasize the left ear width of females, which is larger than that of men in our population, which did not appear in other ethnicities.

The precise measurement of distances between anatomical landmarks in the ear, such as the tragus, helix, and antihelix, plays a pivotal role not only in determining aesthetic features and contours of the pinna but also in the context of reconstructive surgeries and the fitting of hearing aids. This aspect has been explored in previous studies, with findings indicating subtle variations, particularly between genders and among different ethnic groups.

In a study conducted in Turkey, gender-based differences were noted in the distances from the tragus to the helix and antihelix. Men exhibited greater distances (2.63 cm and 1.72 cm, respectively) compared to women (2.51 ± 0.2 cm and 1.66 ± 0.2 cm, respectively) [14]. Similarly, a Sudanese study identified a significant difference between males and females in the distance from the tragus to the antihelix, measuring 1.867 cm and 1.760 cm, respectively [20]. Conversely, a study in Thailand found no significant gender-based variations in these measurements [15].

Our study, however, did not reveal statistically significant differences in the tragus-to-helix distance between sexes. Conversely, the distance from the tragus to the antihelix was observed to be greater in females, with a mean of 2.612 ± 1.2913 cm compared to a mean of 1.903 ± 0.4045 cm among males. This disparity in our findings may be attributed to ethnic and sexual dimorphic factors.

Notably, our research delved into aspects not previously explored in the literature, such as the nose-to-ear distance, which holds significance in plastic surgery procedures addressing ear malformations. We observed a significant difference in this distance between genders for the right ear, where males had a larger nose-to-ear distance (13.160 ± 0.9715 cm) compared to females (12.330 ± 0.5282 cm). No significant difference was found for the left ear, suggesting potential asymmetry.

Comparison with existing literature revealed variations in values, which we attribute to factors such as race, genetic diversity, individual physiology, age, and differing measurement methodologies. Our gender-based analysis indicates overall similarities in measurements, except for specific parameters such as ear lobule width, tragus-antihelix distance, ear width, and the distance from nasion to ear.

Limitations and recommendations

The study sample was limited to the eastern province of the Saudi population. Therefore, the findings of this study may not be indicative of the overall Saudi population, although most of the country’s current population of the country is made up of Arabs. More research should be done by choosing volunteers from different geographic locations and ethnicities in Saudi Arabia. Furthermore, age plays an important role in the morphology of the ears. Moreover, the shape changes with age. Our study included people 20 years of age and older. Further research should be done by recruiting volunteers from different age groups.

## Conclusions

This study provides detailed information about the dimensions of different morphometric parameters of the human ear among the population of Saudi Arabia in the eastern province. Additionally, our findings confirmed the presence of sexual dimorphism among the different ethnic groups, which was also observed in other studies. The anthropometric measurements of the ear in this study also provide a baseline for different studies that can be conducted by anatomists. The study findings can also be used by plastic surgeons for different surgical procedures related to the ear such as ear reconstruction and implantations of ear prosthetics. This data can also be used by forensic experts for further studies related to facial recognition, anthropometry, etc. The morphometric details of the human ear presented in this study can also be useful for designing hearing aids, prostheses, and implants. Further research can be conducted using larger sample sizes including volunteers from different regions of the country to establish a normal range of these parameters for Saudi Arabia.
